# Supplementary material for: Structural plasticity of SARS-CoV-2 3CL Mpro active site cavity revealed by room temperature X-ray crystallography
Source: Nat Commun. 2020 Jun 24;11:3202. doi: 10.1038/s41467-020-16954-7 (PMC7314768; doi:10.1038/s41467-020-16954-7)
Supplement: Supplementary file 1 — Supplementary Information [file 41467_2020_16954_MOESM1_ESM.pdf]

Supplementary Information

Structural Plasticity of SARS-CoV-2 3CL M^{pro} Active Site Cavity Revealed by Room Temperature X-ray Crystallography

Daniel W. Kneller,[1] Gwyndalyn Phillips,[1] Hugh M. O'Neill,[1] Robert Jedrzejczak,[2,3] Lucy Stols,[2] Paul Langan,[1] Andrzej Joachimiak,[2,3,4] Leighton Coates,[1*] and Andrey Kovalevsky,[1*]

Supplementary Table 1. DNA sequence for the codon-optimized gene of SARS-CoV-2 3CL M^{pro}.

AGCGCTGTTCTGCAGTCTGGTTTCCGTAAAATGGCTTTCCCGTCTGGTAAAGTTGAA
GGTTGTATGGTTCAGGTAACCTGCGGCACTACCACCCTGAACGGCCTGTGGCTGGAT
GACGTTGTTTACTGCCCGCGTCATGTTATCTGTACTTCCGAAGATATGCTGAACCCG
AACTACGAAGATCTGCTGATCCGTAAATCTAACCACAACTTCCTGGTTCAGGCAGGT
AACGTTCAGCTGCGTGTTATCGGTCACTCTATGCAGAACTGCGTTCTGAAACTGAAA
GTTGATACCGCTAACCCGAAAACCCCGAAATACAAATTCGTTCGTATCCAGCCGGGT
CAGACCTTCAGCGTTCTGGCTTGCTATAACGGTTCTCCGTCTGGTGTTTACCAGTGCG
CTATGCGTCCGAACTTCACTATTAAAGGTTCCTTCCTGAACGGTTCTTGTGGTTCTGT
TGGTTTCAACATTGATTACGATTGCGTTTCTTTCTGCTACATGCACCACATGGAACTG
CCGACTGGTGTTCACGCTGGCACCGATCTGGAAGGTAACTTTTACGGTCCGTTCGTT
GACCGTCAGACCGCTCAGGCTGCTGGTACTGATACCACCATTACCGTTAACGTTCTG
GCTTGGCTGTACGCTGCTGTTATCAACGGTGATCGTTGGTTCCTGAACCGTTTCACCA
CCACCCTGAACGATTTCAACCTGGTTGCGATGAAATACAACTACGAACCGCTGACCC
AGGATCACGTTGACATCCTGGGTCCGCTGTCTGCTCAGACCGGTATCGCTGTTCTGG
ATATGTGCGCTTCTCTGAAAGAACTGCTGCAGAACGGTATGAACGGTCGTACCATCC
TGGGTTCTGCTCTGCTGGAAGATGAATTTACCCCGTTCGATGTTGTTCGTCAGTGCTC
TGGTGTTACCTTCCAGGGTCCGCATCATCACCATCACCAT

Supplementary Table 2. Data reduction and refinement statistics for the room temperature structure of the 3CL M^{pro} from SARS-CoV-2.

	**3CL M^{pro}**, PDB ID 6WQF
**Data collection:**	**X-ray**
Diffractometer	Rigaku HighFlux Eiger 4M
Space group	I2
Wavelength (Å)	1.54
Cell dimensions:	
a, *b*, *c* (Å)	45.07, 54.06, 113.61
α, β, γ (°)	90, 100.51, 90
Resolution (Å)	27.92-2.30 (2.38-2.30)*
No. reflections unique	11514 (858)
R_{merge}	0.110 (0.621)
R_{pim}	0.065 (0.421)
$CC_{1/2}$	0.993 (0.666)
I / σI	8.1 (1.9)
Completeness (%)	95.4 (72.6)
Redundancy	3.7 (2.7)

**Refinement:**	**X-ray**
Resolution	27.92 – 2.30
R_{work} / R_{free}	0.180 / 0.230
Ramachandran statistics	
Favored (%)	95.72
Allowed (%)	4.28
Outliers (%)	0
R.M.S. deviations	
Bond lengths (Å)	0.002
Bond angles (°)	0.444
All atom clashscore	0.85
B-factors (Å^2)	
Protein (overall)	37.1
Protein (main chain)	35.4
Protein (side chain)	38.9
Water	32.6

* Values in parentheses are for highest-resolution shell. Data were collected from one crystal.

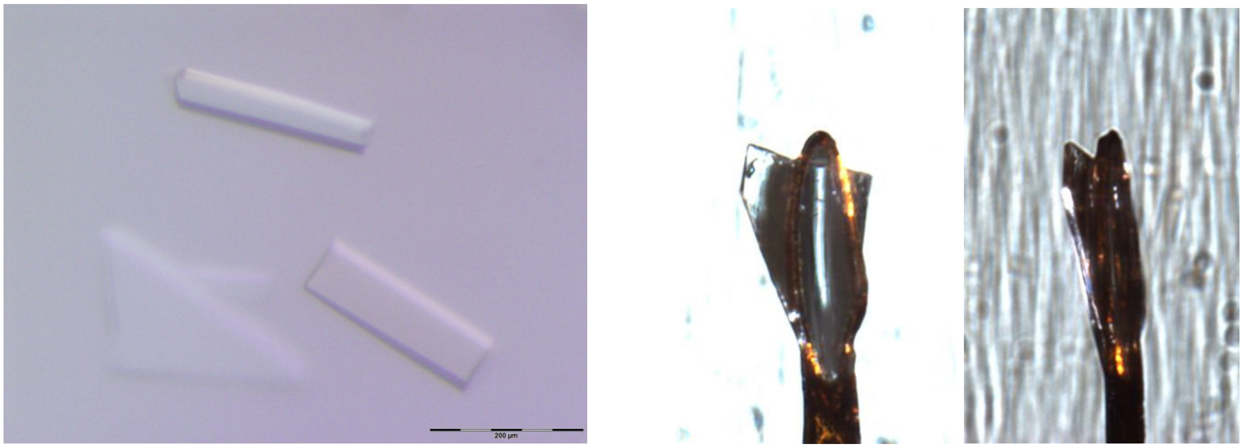

A **B** **C**

Supplementary Figure 1. (**A**) Crystals of 3CL M^{pro} grown using the microseeding technique; (**B, C**) Crystal mounted on a loop used for the room-temperature X-ray data collection.

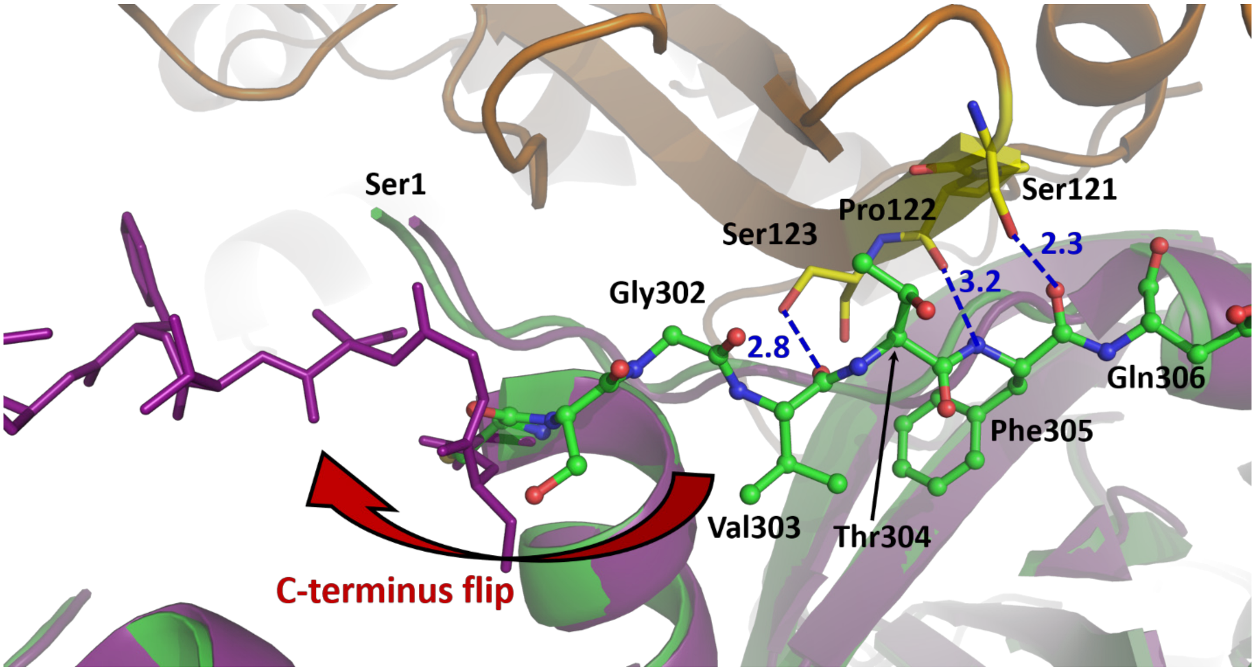

Supplementary Figure 2. Superposition of the room temperature ligand-free structure of 3CL M^{pro} (green carbon atoms) in complex with inhibitor N3 (deep purple, PDB ID 6LU7) from SARS-CoV-2. The C-terminal tail flips 180 ° upon the inhibitor binding (shown by the curved red arrow).

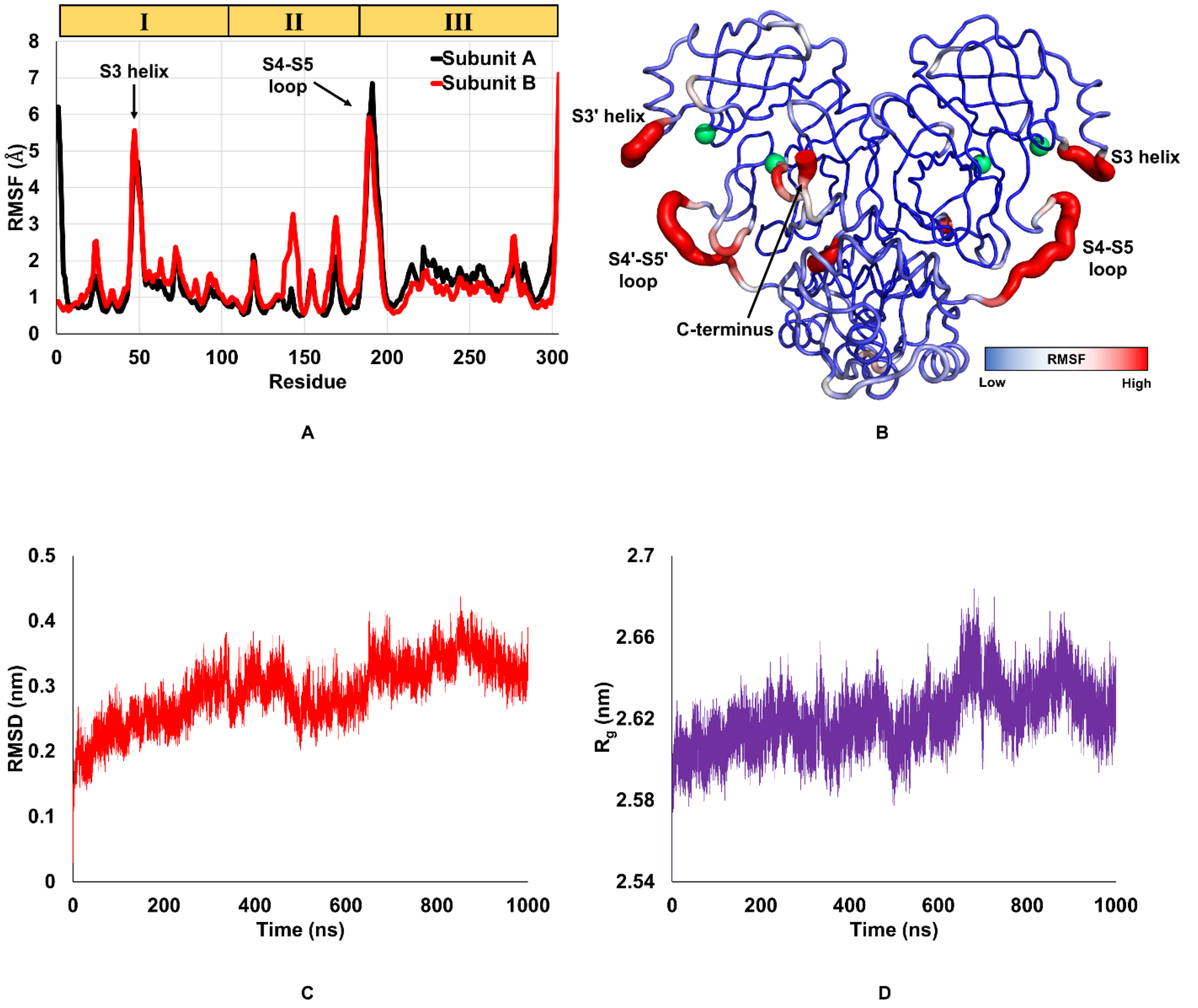

Supplementary Figure 3. Root Mean Square Fluctuation (RMSF) analysis from a 1 µs MD simulation of an apo 3CL M^{pro} dimer. (**A**) Protein backbone RMSF by residue for Subunits A and B. (**B**) Cartoon-putty representation of 3CL M^{pro} colored by protein backbone RMSF with Cα of His41/41′ and Cys145/145′ shown as green spheres. (**C**) RMSD of backbone atoms relative to the equilibrated structure. (**D**) Radius of gyration (R_g = 26.2 ± 0.15 Å) of 3CL M^{pro} dimer.
